# Supplementary material for: Gut microbiome dysbiosis across early Parkinson’s disease, REM sleep behavior disorder and their first-degree relatives
Source: Nat Commun. 2023 May 2;14:2501. doi: 10.1038/s41467-023-38248-4 (PMC10154387; doi:10.1038/s41467-023-38248-4)
Supplement: Supplementary file 1 — Supplementary information [file 41467_2023_38248_MOESM1_ESM.pdf]

Supplementary Information

Gut microbiome dysbiosis across early Parkinson's disease, REM sleep behavior disorder and their first-degree relatives

Bei Huang, Steven WH Chau, Yaping Liu, Joey WY Chan, Jing Wang, Suk Ling Ma, Jihui Zhang, Paul KS Chan, Yun Kit Yeoh, Zigui Chen, Li Zhou, Sunny Hei Wong, Vincent CT Mok, Ka Fai To, Hei Ming Lai, Simon Ng, Claudia Trenkwalder, Francis KL Chan & Yun Kwok Wing*

* Correspondence: Dr. Yun Kwok Wing, Professor, Director of Li Chiu Kong Family Sleep Assessment Unit, Department of Psychiatry, Shatin Hospital, The Chinese University of Hong Kong, Shatin, Hong Kong SAR. Tel: 852-39197748. Fax: 852-26475321. E-mail: ykwing@cuhk.edu.hk

Inventory of Supporting Information

Supplementary Figure 1	Summary of the inclusion and exclusion criteria for each stage of α-synucleinopathy	Page 3
Supplementary Figure 2	Additional information on overall microbial composition analysis.	Page 5
Supplementary Figure 3	The abundance of *[Eubacterium]_ventriosum_group* (a) and *Collinsella* (b) between controls, RBD-FDR with and without probable RBD	Page 7
Supplementary Figure 4	Changes of differential family at prodromal and early α-synucleinopathy	Page 8
Supplementary Figure 5	Machine learning analysis framework	Page 9
Supplementary Figure 6	Random Forest model differentiating RBD from RBD-FDR.	Page 10
Supplementary Figure 7	Spearman correlation of abundances between differential genera and metabolic pathways.	Page 11
Supplementary Figure 8	Shifted overall microbial composition in the subgroups of RBD patients.	Page 14
Supplementary Figure 9	Understanding the interactions of microbiota with physical activity (a) and antidepressant use (b)	Page 15

Supplementary Figures

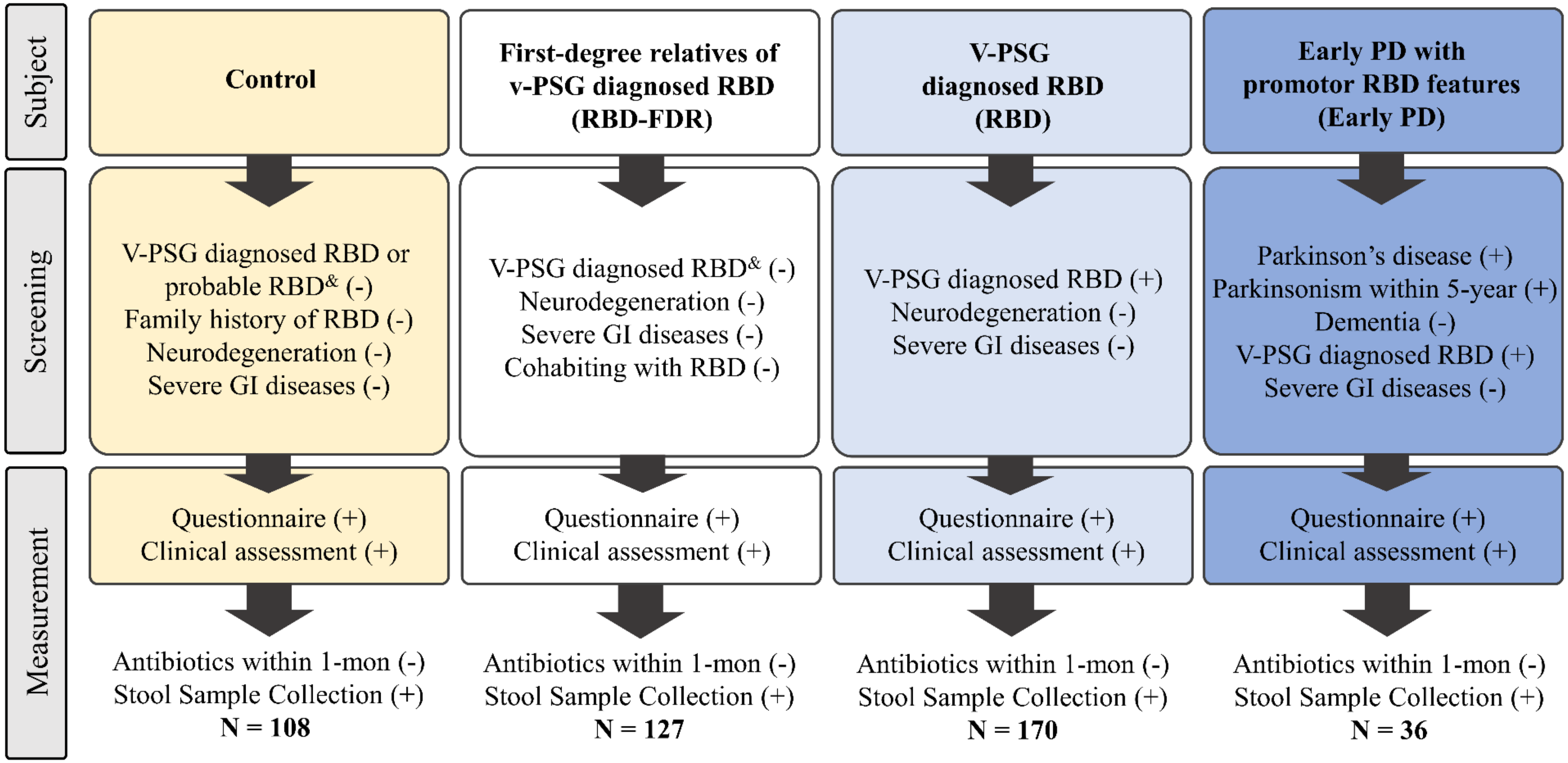

Supplementary Figure 1 Summary of the inclusion and exclusion criteria for each stage of α-synucleinopathy. This figure summarizes the inclusion and exclusion criteria for control, RBD-FDR, RBD and early PD. Video-polysomnography (v-PSG) is the gold standard for diagnosing RBD. When v-PSG was not feasible, a diagnosis of probable RBD was made based on the clinical assessment, namely the Diagnostic Interview for Sleep Patterns and Disorders. Dementia was defined as a total score of Hong Kong version of

Montreal Cognitive Assessment (HK-MoCA) ≤ 21 and Clinical Dementia Rating ≥ 1. In this figure, (-) and (+) correspond to exclusion and inclusion criterion, respectively. [&] V-PSG was assessed in a subset of control (n = 64) and RBD-FDR (n = 91) subjects. RBD, REM sleep behavior disorder; RBD-FDR, first-degree relatives of RBD patients; PD, Parkinson's disease; GI, gastrointestinal; v-PSG, video-polysomnography.

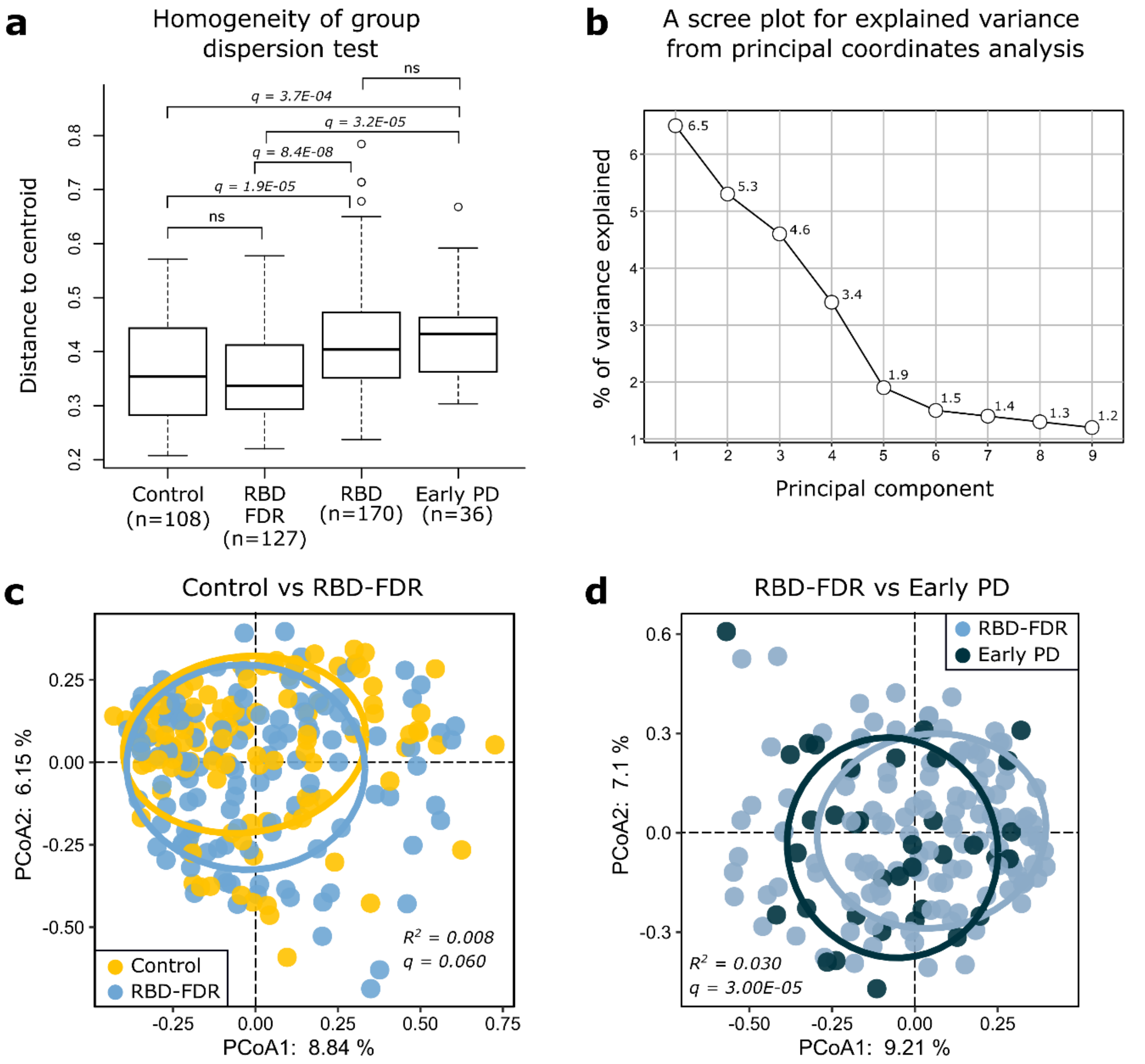

Supplementary Figure 2 Additional information on overall microbial composition analysis. a, Boxplot depicts intra-group Bray-Curtis distance to group centroid. Statistical differences were analyzed using Kruskal-Wallis test with post-hoc analysis, p values for multiple testing were adjusted applying Benjamini-Hochberg method. All box plots represented the interquartile interval of the data, with the median shown as a thick line in the middle of the box; whiskers extend up to values within 1.5 times of interquartile range, and outliers are plotted as individual points beyond the whiskers. **b,** Scree plot for explained variance of first nine principal components from the principal coordinates analysis. Principal Component was displayed on the x axis and percentage of variance explained was shown on the y axis. **c-d,** Principal coordinates analysis of microbial communities between groups with 70% confidence ellipse. Significance of inter-group differences in overall microbial

composition was calculated by PERMANOVA (two-sided test) with adjustment of age and sex (permutation = 99,999). R^2 indicated the inter-individual variation explained by grouping factors in PERMANOVA test. RBD, REM sleep behavior disorder; RBD-FDR, first-degree relatives of patients with RBD; PD, Parkinson's disease; PERMANOVA, permutational multivariate analysis of variance; ns, not significant. Source data are provided as a Source Data file.

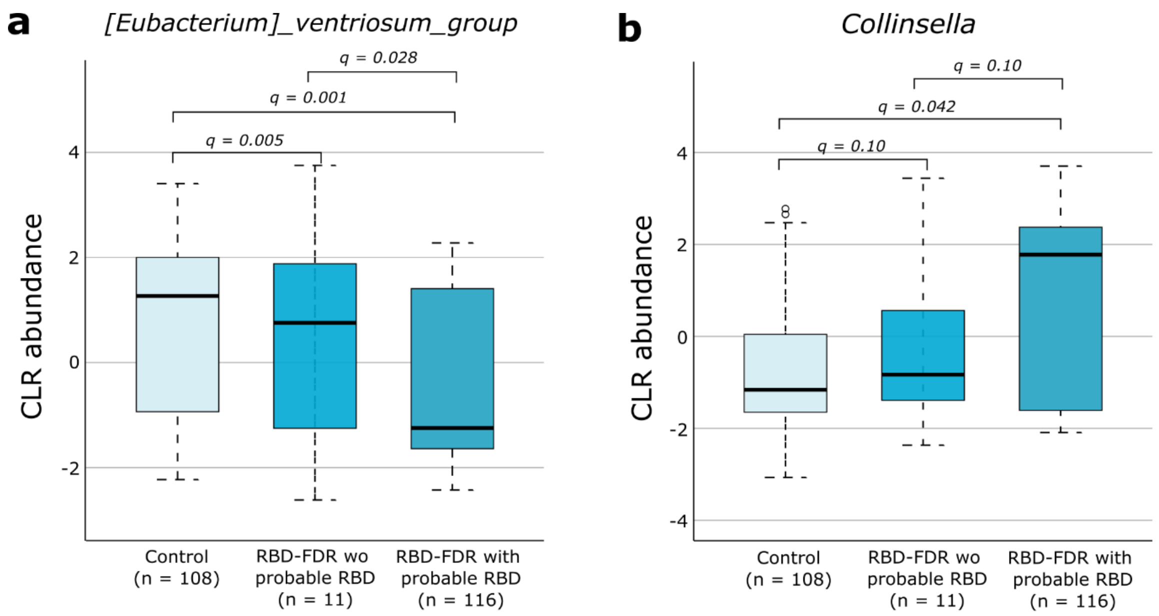

Supplementary Figure 3 The abundance of *[Eubacterium]_ventriosum_group* (a) and *Collinsella* (b) between controls, RBD-FDR with and without probable RBD. Boxplot depicts the centered log-ratio abundance of two differential genera *[Eubacterium]_ventriosum_group* (**a**) and *Collinsella* (**b**) in control (n = 108), RBD-FDR with (n = 11) and without probable RBD (n = 116). Probable RBD was diagnosed according to the Diagnostic Interview for Sleep Patterns and Disorders (DISP). The box covers the interquartile interval of the data, with the median shown as a thick line in the middle of the box; whiskers extend up to values within 1.5 times of interquartile range; outliers are plotted as individual points beyond the whiskers. Statistical differences were analyzed using Generalized Estimating Equations model (two-sided test) with adjustment of age and sex, p values for multiple testing were adjusted applying Benjamini-Hochberg method. RBD, REM sleep behavior disorder; RBD-FDR, first-degree relatives of patients with RBD; CLR, centered log-ratio. Source data are provided as a Source Data file.

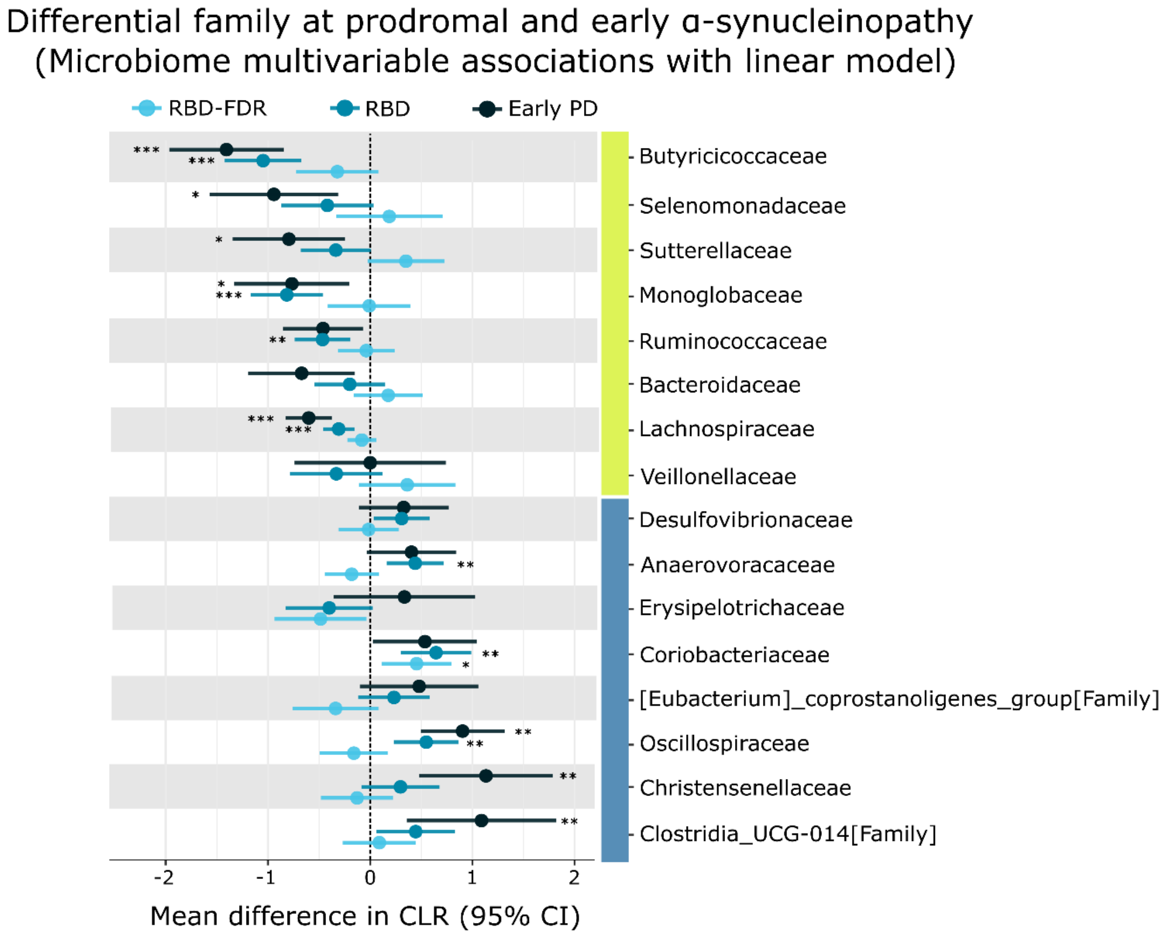

Supplementary Figure 4 Changes of differential family at prodromal and early α-synucleinopathy. This is a supplementary figure for main text Fig. 3b. Error bar plot demonstrates mean difference of differential family at prodromal and early α-synucleinopathy as compared with control, as well as 95% confidence interval of the mean difference. The blue and yellow bars along the vertical axis of the plot indicated taxa increased and decreased with disease progression (Kendall's τb > 0 and < 0, respectively). Significance of changes were analysed using MaAsLin 2. The model was fitted with group as fixed effect, family clustering as random effect, and without other adjustment of covariates. "***", "**" and "*" represent Benjamini-Hochberg method adjusted p values (q-values) less than 0.001, 0.01, and 0.05, respectively. RBD, REM sleep behavior disorder; RBD-FDR, first-degree relatives of patients with RBD; PD, Parkinson's disease; MaAsLin 2, Microbiome Multivariable Associations with Linear Model; CLR, centered log-ratio. Source data are provided as a Source Data file.

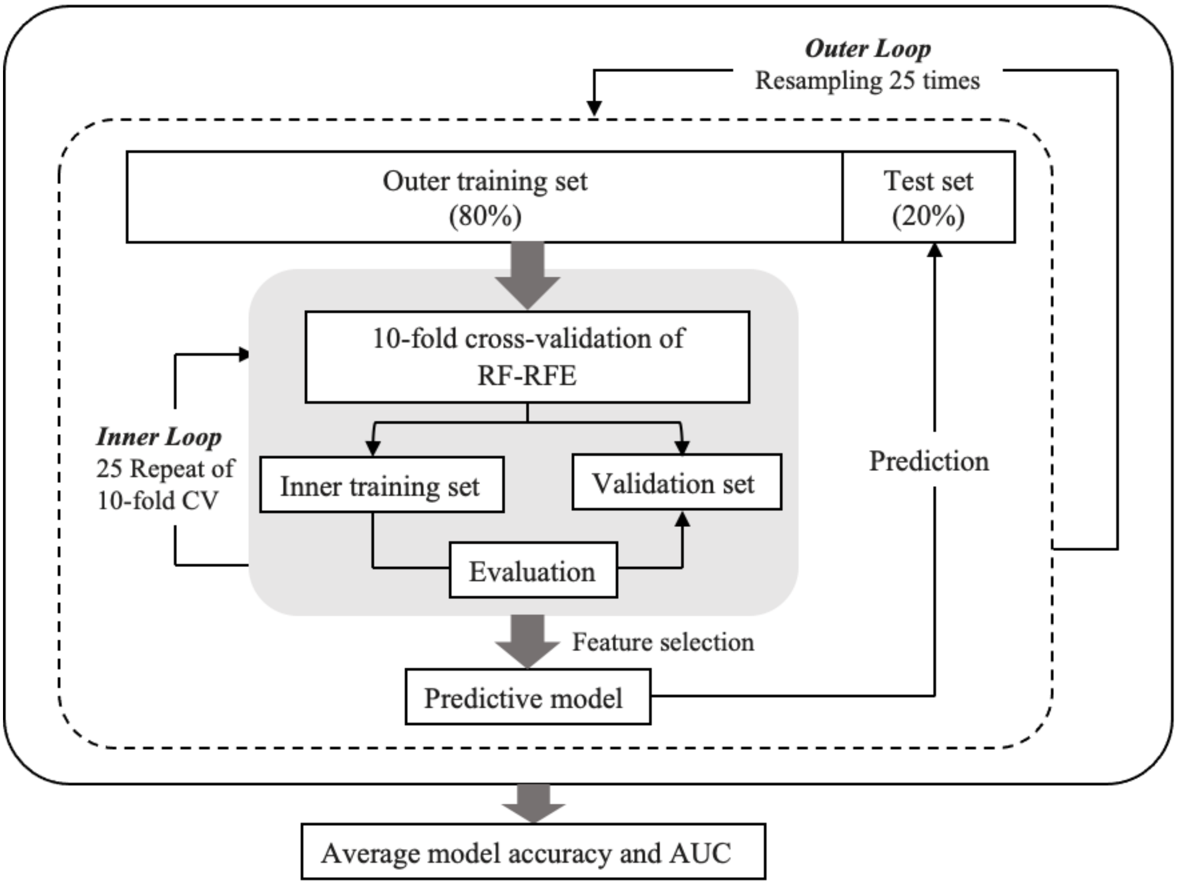

Supplementary Figure 5 Machine learning analysis framework. For each random forest classification model, the original dataset was randomly partitioned into an outer training set (80%) and an independent test set (20%). Within the outer training set, the data were further split into inner training set and validation set for feature selection ("Inner loop", 25 repeats of 10-fold cross-validation) based on the recursive feature elimination algorithm. The final model was trained with the selected features and its performance were examined in the test set. The whole process of feature selection, final model training, and performance testing was repeated 25 times (i.e., "Outer loop"), and the average performance estimates (e.g., accuracy and area under the curve) would be reported. RF, random forest; RFE, recursive feature elimination; CV, cross-validation; AUC, area under the operating characteristic curve.

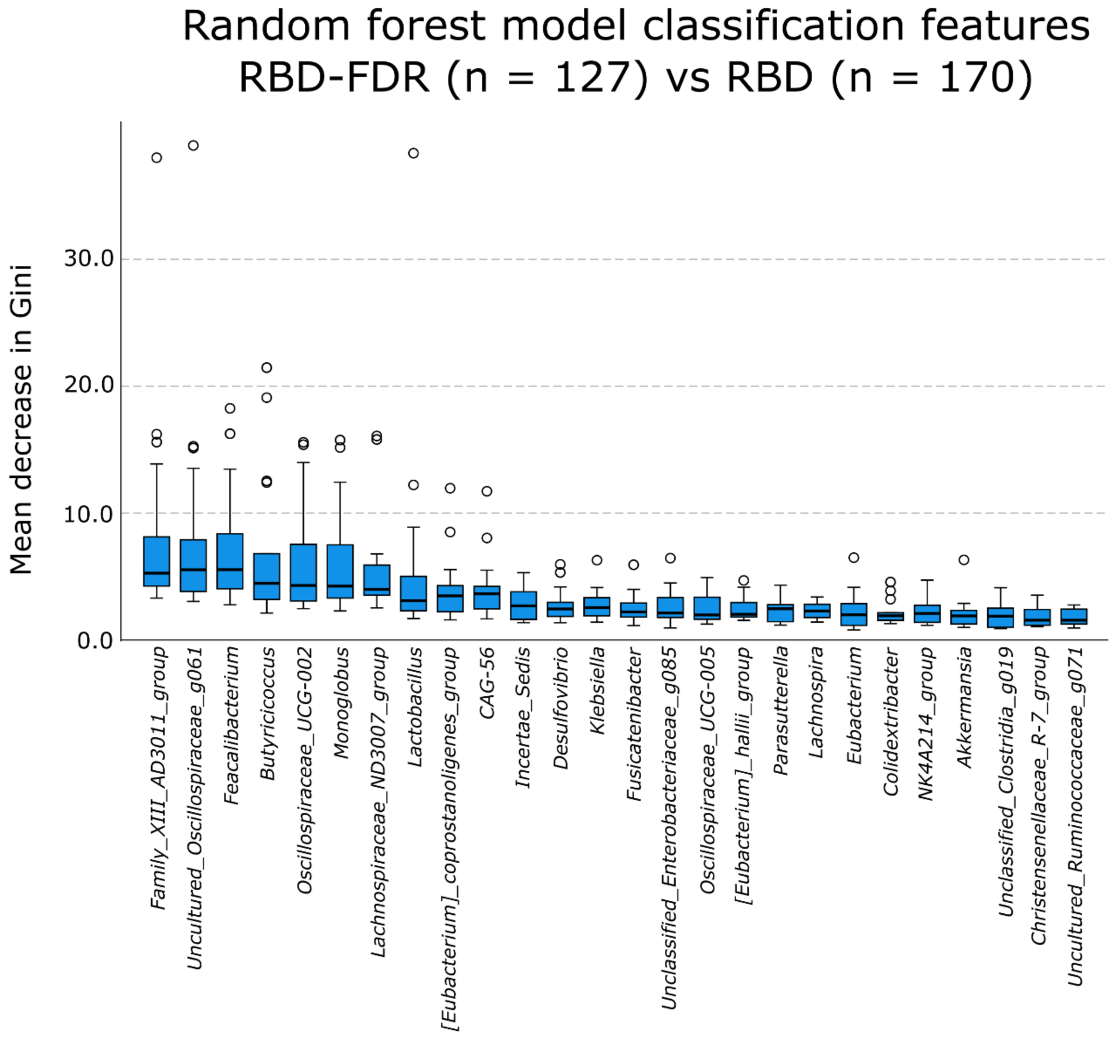

Supplementary Figure 6 Random Forest model differentiating RBD from RBD-FDR. Boxplot showed the results of feature selection in random forest model differentiating RBD (n = 170) from RBD-FDR (n = 127). Microbial markers appeared in at least 60% of all 25 final trained models were considered as classification features. The box covers the interquartile interval of the data, with the median shown as a thick line in the middle of the box; whiskers extend up to values within 1.5 times of interquartile range, and outliers are plotted as individual points beyond the whiskers. Source data are provided as a Source Data file.

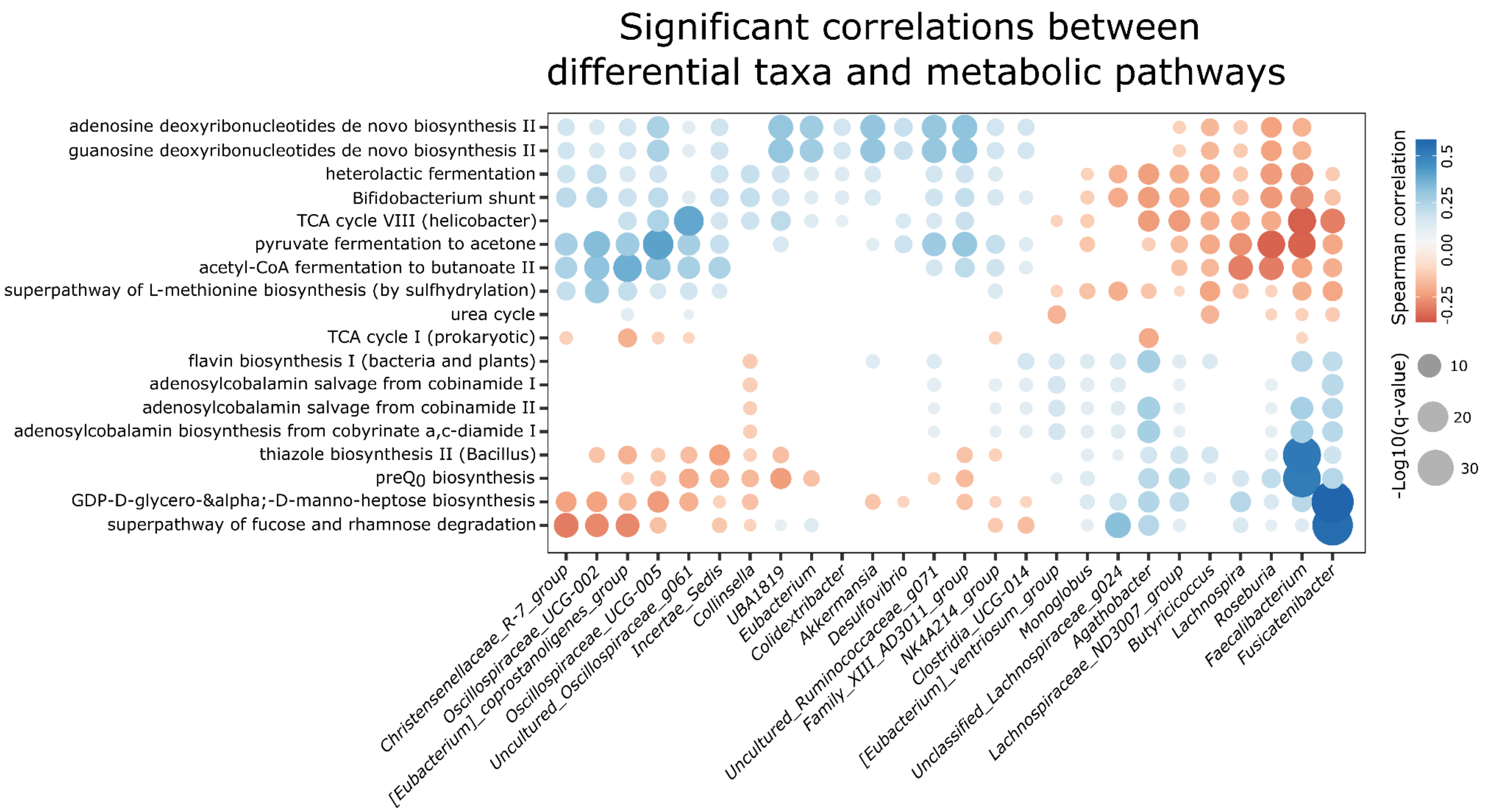

Supplementary Figure 7 Spearman correlation of abundances between differential genera and metabolic pathways. The associations between differential genera (n = 26) and metabolic pathways (n = 18) among all samples. Only significant correlations (q-value < 0.05) derived from Spearman

correlation analysis (two-sided test) were shown in the heatmap. The color and size of the node indicate the Spearman coefficient and the negative value of log-transformed q value, respectively. Source data are provided as a Source Data file.

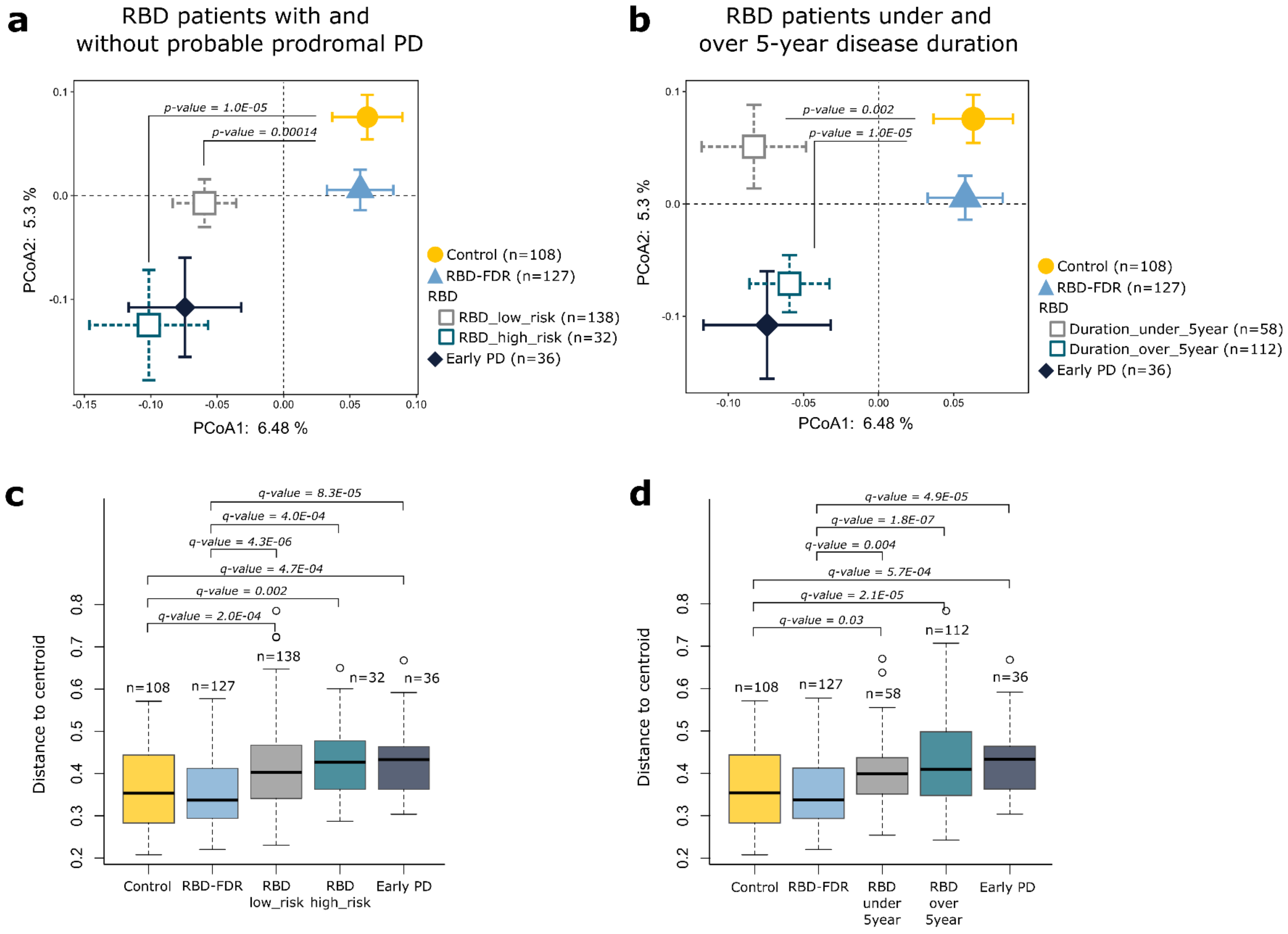

a
RBD patients with and without probable prodromal PD
p-value = 1.0E-05
p-value = 0.00014
PCoA2: 5.3 %
PCoA1: 6.48 %
Control (n=108)
RBD-FDR (n=127)
RBD
RBD_low_risk (n=138)
RBD_high_risk (n=32)
Early PD (n=36)
b
RBD patients under and over 5-year disease duration
p-value = 0.002
p-value = 1.0E-05
PCoA2: 5.3 %
PCoA1: 6.48 %
Control (n=108)
RBD-FDR (n=127)
RBD
Duration_under_5year (n=58)
Duration_over_5year (n=112)
Early PD (n=36)
c
q-value = 8.3E-05
q-value = 4.0E-04
q-value = 4.3E-06
q-value = 4.7E-04
q-value = 0.002
q-value = 2.0E-04
Distance to centroid
n=108
n=127
n=138
n=32
n=36
Control
RBD-FDR
RBD low_risk
RBD high_risk
Early PD
d
q-value = 4.9E-05
q-value = 1.8E-07
q-value = 0.004
q-value = 5.7E-04
q-value = 2.1E-05
q-value = 0.03
Distance to centroid
n=108
n=127
n=58
n=112
n=36
Control
RBD-FDR
RBD under 5year
RBD over 5year
Early PD

Supplementary Figure 8 Shifted overall microbial composition in the subgroups of RBD patients. a-b, Principal coordinates analysis (PCoA) of microbial communities across the early stages of α-synucleinopathy based on Bray-Curtis distance matrix at the genus level. Statistical differences between RBD subgroups and control were analyzed using PERMANOVA with adjustment of age and sex (permutation = 99,999, two-sided test). The position of each group was the average (i.e., group centroid) of PCoA1 (x axis) and PCoA2 (y axis). Horizontal and vertical error bars indicated the standard errors of the averages. **c-d,** Boxplot depict intra-group Bray-Curtis distance to group centroid. Statistical differences were analyzed using Kruskal-Wallis test with post-hoc analysis (post hoc multiple comparisons were adjusted by Benjamini-Hochberg method). The box covers the interquartile interval of the data, with the median shown as a thick line in the middle of the box; whiskers extend up to values within 1.5 times of interquartile range; outliers are plotted as individual points beyond the whiskers. Only significant differences (q values < 0.05) are shown. RBD, REM sleep behavior disorder; RBD-FDR, first-degree relatives of patients with RBD; PD, Parkinson's disease; PERMANOVA, permutational multivariate analysis of variance. Source data are provided as a Source Data file

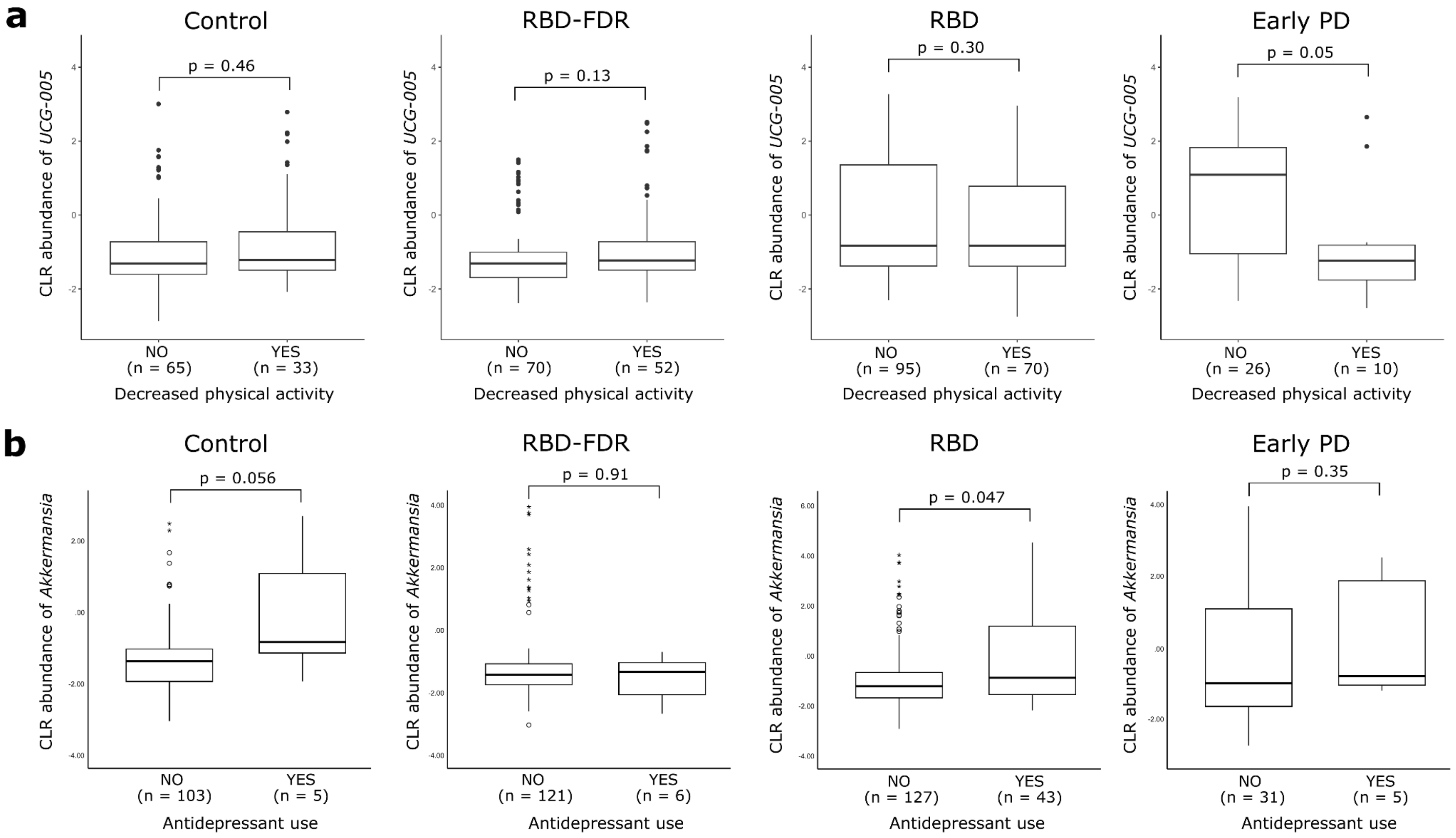
a
Control
RBD-FDR
RBD
Early PD
p = 0.46
p = 0.13
p = 0.30
p = 0.05
CLR abundance of UCG-005
NO
(n = 65)
YES
(n = 33)
NO
(n = 70)
YES
(n = 52)
NO
(n = 95)
YES
(n = 70)
NO
(n = 26)
YES
(n = 10)
Decreased physical activity
b
Control
RBD-FDR
RBD
Early PD
p = 0.056
p = 0.91
p = 0.047
p = 0.35
CLR abundance of Akkermansia
NO
(n = 103)
YES
(n = 5)
NO
(n = 121)
YES
(n = 6)
NO
(n = 127)
YES
(n = 43)
NO
(n = 31)
YES
(n = 5)
Antidepressant use

Supplementary Figure 9 Understanding the interactions of microbiota with physical activity (a) and antidepressant use (b). a, the distributions of *UCG-005* (family Oscillospiraceae) abundance between subjects with (YES) and without (NO) decreased physical activity (i.e., moderate-to-vigorous physical activity less than one hour per week). Group differences were analyzed using two-sided Mann–Whitney U test. N indicates the number of subjects in different groups classified as YES or NO. The box covers the interquartile interval of the data, with the median shown as a thick line in the middle of the box; whiskers extend up to values within 1.5 times of interquartile range; outliers are plotted as individual points beyond the whiskers. **b,** The distributions of genus *Akkermansia* abundance between subjects with (YES) and without (NO) antidepressant use. Group differences were analyzed using two-sided Mann–Whitney U test. N indicates the number of subjects in different groups classified as YES or NO. The box covers the interquartile interval of the data, with the median shown as a thick line in the middle of the box; whiskers extend up to values within 1.5 times of interquartile range; outliers are plotted as individual points beyond the whiskers. RBD, REM sleep behavior disorder; RBD-FDR, first-degree relatives of patients with RBD; PD, Parkinson’s disease; CLR, centered log-ratio. Source data are provided as a Source Data file.
