# Supplementary material for: Gut microbiome dysbiosis across early Parkinson’s disease, REM sleep behavior disorder and their first-degree relatives
Source: Nat Commun. 2023 May 2;14:2501. doi: 10.1038/s41467-023-38248-4 (PMC10154387; doi:10.1038/s41467-023-38248-4)
Supplement: Supplementary file 3 — Description of Additional Supplementary Files [file 41467_2023_38248_MOESM3_ESM.pdf]

Description of Additional Supplementary Files

File Name: Supplementary Dataset 1

Description: Sociodemographic, gastrointestinal, and clinical characteristics

File Name: Supplementary Dataset 2

Description: Analysis of alpha-diversity between groups

File Name: Supplementary Dataset 3

Description: Pairwise comparisons of overall microbial composition

File Name: Supplementary Dataset 4

Description: Interactions between overall microbial composition and host factors

File Name: Supplementary Dataset 5

Description: Differential genera across prodromal and early stages of α-synucleinopathy

File Name: Supplementary Dataset 6

Description: Correlations of first two principal components and genera abundance

File Name: Supplementary Dataset 7

Description: Differential family across prodromal and early stages of α-synucleinopathy

File Name: Supplementary Dataset 8

Description: Associations of different stages of α-synucleinopathy and taxa abundance (unadjusted model)

File Name: Supplementary Dataset 9

Description: Associations of different stages of α-synucleinopathy and taxa abundance (adjusted model)

File Name: Supplementary Dataset 10

Description: Associations of host factors and taxa abundance

File Name: Supplementary Dataset 11

Description: The effect of PD specific drugs on taxa and pathway abundance

File Name: Supplementary Dataset 12

Description: Differential metabolic pathways across prodromal and early stages of α-synucleinopathy

File Name: Supplementary Dataset 13

Description: Associations of different stages of α-synucleinopathy and pathway abundance (unadjusted model)

File Name: Supplementary Dataset 14

Description: Associations of different stages of α-synucleinopathy and pathway abundance (adjusted model)

File Name: Supplementary Dataset 15

Description: Associations of host factors and pathway abundance

File Name: Supplementary Dataset 16

Description: Results of random forest classification
